# *p*-Value Histograms: Inference and Diagnostics

**DOI:** 10.3390/ht7030023

**Published:** 2018-08-31

**Authors:** Patrick Breheny, Arnold Stromberg, Joshua Lambert

**Affiliations:** 1 Department of Biostatistics, University of Iowa, Iowa City, IA 52242, USA; patrick-breheny@uiowa.edu; 2 Department of Statistics, University of Kentucky, Lexington, KY 40508, USA; stromberg@uky.edu

**Keywords:** *p*-value, histograms, inference, diagnostics

## Abstract

It is increasingly common for experiments in biology and medicine to involve large numbers of hypothesis tests. A natural graphical method for visualizing these tests is to construct a histogram from the *p*-values of these tests. In this article, we examine the shapes, both regular and irregular, that these histograms can take on, as well as present simple inferential procedures that help to interpret the shapes in terms of diagnosing potential problems with the experiment. We examine potential causes of these problems in detail, and discuss potential remedies. Throughout, examples of irregular-looking *p*-value histograms are provided and based on case studies involving real biological experiments.

## 1. Introduction

**Figure 1:**
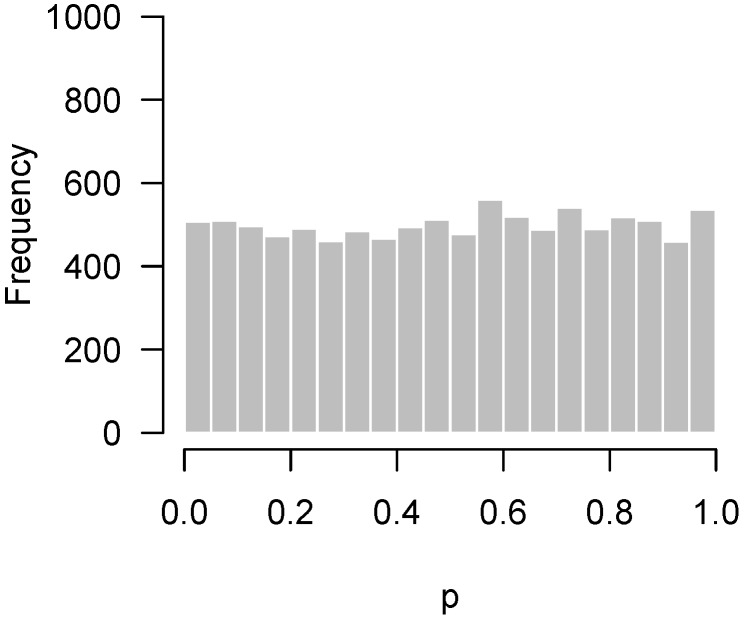
Simulated *p*-values from an idealized setting in which all null hypotheses are true.

Since the advent of high-throughput technology, it has become common for experiments in biology and medicine to involve large numbers of hypothesis tests. A natural graphical method for visualizing the body of these tests is to take the *p*-values from these tests and construct a histogram. If all null hypotheses are true, these *p*-values follow a uniform distribution, which corresponds to a flat-looking histogram. Figure 1 illustrates an idealized version of this histogram in which 10,000 *p*-values have been drawn from a uniform random number generator.

**Figure 2:**
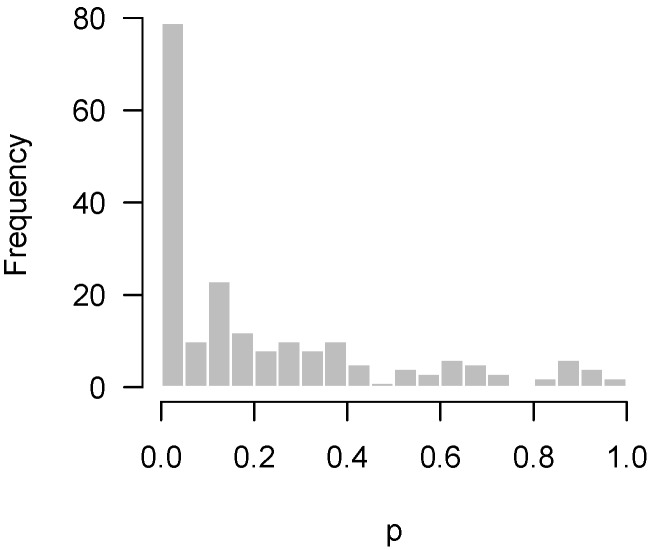
Rogier et al. [1]: Overabundance of low *p*-values in a sufficiently powered experiment.

Of course, one typically hopes that some of these null hypotheses are incorrect, and that there is an overabundance of low *p*-values. For example, in Rogier et al. [1], 201 genes were tested for differential expression upon exposure to azoxymethane (AOM) and dextran sulfate sodium (DSS) in neonatal mice using nanostring hybridization. Two sample *t*-tests were applied to each gene; the resulting *p*-values are displayed in Figure 2. In the histogram, the *p*-values appear to be relatively uniform except for the clear overabundance of very low *p*-values.

There has been a tremendous amount of work in the past two decades, in particular involving false discovery rates [2], extending multiple comparison procedures to large-scale simultaneous inference questions such as these. Naturally, the vast majority of this work has focused on questions involving individual hypotheses. Our focus here, however, concerns what the *p*-value histogram says about the experiment as a whole. Some examples help to illustrate what we mean by this.

**Figure 3:**
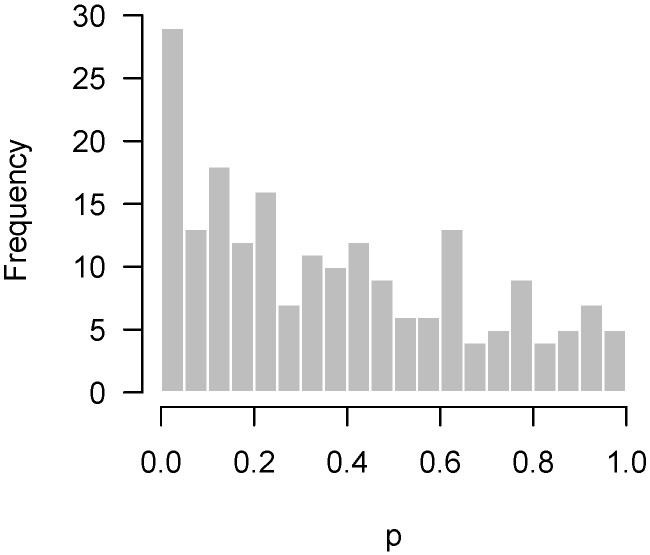
Rogier et al. [1]: Overabundance of low *p*-values in an insufficiently powered experiment.

Figure 3 displays another set of *p*-values, also from Rogier et al. [1], this time involving mice which were not injected with AOM. In the experiment, not a single hypothesis could be rejected at the 10% false discovery rate level. However, as we can see in the figure, the *p*-values clearly do not seem to be uniformly distributed. There is an apparent overabundance of low *p*-values, suggesting the existence of genes in mice that genuinely responded to DSS. However, the experiment is not sufficiently powered to detect them after making corrections for multiple testing.

**Figure 4:**
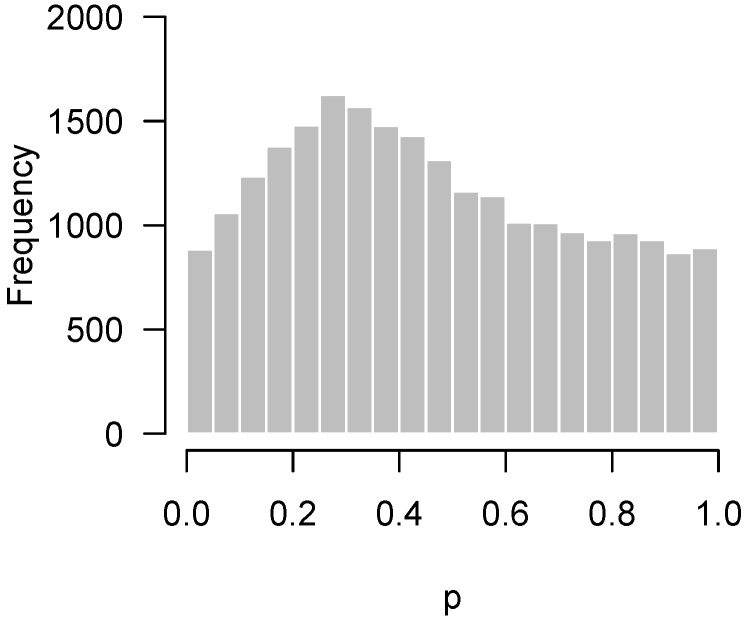
Fischl et al. [3]: Overabundance of *p*-values near 0.3. Possible problematic experiment.

Lastly, Figure 4 presents the *p*-values of an experiment by Fischl et al. [3] where paired *t*-tests were used to compare dependent samples. From the histogram, it appears as though something has gone wrong: there is an abundance not of low *p*-values but of *p*-values near 0.3. In summary, we have encountered four examples: no interesting features to detect (Figure 1); interesting features easily detected (Figure 2); interesting features present but unable to be detected (Figure 3); and finally, a problematic experiment (Figure 4). We discuss these cases in greater detail below and provide diagnostics for distinguishing between them. Throughout this manuscript, we use the generic term “feature” to refer to the quantity being measured in a high-throughput experiment; in our examples, the features are gene expression levels, but all of the ideas in the article are equally applicable to any high-throughput measurement such as metabolite or protein concentrations.

All histograms were produced in R (www.r-project.org). The code that was used to produce them can be found in the Appendix A.

## 2. Methods

### 2.1. Background

This section provides a brief background on hypothesis testing with multiple comparisons, and defines several terms that will be used throughout the paper. For a *p*-value to be valid, a *p*-value must satisfy Pr(p<α)≤α under the null. For the sake of simplicity, we mostly assume we are working with proper *p*-values, in which Pr(p<α)=α under the null hypothesis and are therefore uniformly distributed.

The above properties mean that, if we declare hypotheses to be false if p<α, the proportion of time we incorrectly reject a truly null hypothesis will be no greater than α; this is known as the type I error rate. When multiple hypotheses are being tested, however, there may be many false positives (type I errors) even if the type I error rate is low. Thus, more strict significance criteria are typically required when multiple comparisons are being made.

The most widespread alternative to the type I error rate for high-throughput hypothesis testing is the false discovery rate (FDR). Rather than limit the fraction of null hypotheses that can be rejected, the false discovery rate limits the fraction of rejected tests that come from null hypotheses. It is unknown, of course, whether a given test is null, thus, in practice, FDR methods instead control the expected value of this fraction. The most widely used procedure for FDR control was developed by Benjamini and Hochberg [2], which is what we use throughout in this manuscript, although we note that there is an extensive amount of literature on the subject with many alternative approaches.

If the hypothesis being tested is not null, then the above framework places no restrictions on what its distribution may look like. In practice, however, we would like the *p*-values for these cases to be as small as possible, and design the experiment in order to maximize the probability of obtaining as many low *p*-values as possible (i.e., the power). Thus, we expect the results of a high-throughput experiment to resemble a combination of a uniform distribution (from the null hypotheses) and a distribution with an overabundance of low *p*-values (from the non-null hypotheses). For the purposes of this paper, we define a “regular” *p*-value histogram to be one that reflects such a combination. In other words, a regular *p*-value histogram is one that is either uniform (Figure 1) or slopes downward left-to-right (as in Figure 2 and Figure 3). We define an “irregular” histogram to be one that has any other shape (for example, Figure 4).

### 2.2. Higher Criticism

For the data presented in Figure 3, not a single null hypothesis could be rejected at a false discovery rate of 5%. However, it seems clear from looking at the histogram that something is going on and that more low *p*-values are present than one would expect by chance alone. This claim can be tested using quantiles of the binomial distribution. Let *b* denote the bin width of the histogram, *m* denote the number of hypotheses being tested, *X* denote the number of *p*-values in the bin closest to zero, and $F_{\alpha }\left(m,p\right)$ denote the α-level quantile of the cumulative distribution function (CDF) of the binomial distribution with size *m* and probability *p*. Then, under the global null hypothesis $H_{0j}:p_{j}∼\mathrm{Unif}\left(0,1\right)$ for all *j*, the probability that *X* exceeds $F_{0.95}\left(m,b\right)$ is only 5%. Note that, arguably, the 0.975 quantile could be used instead, as it would be consistent with the standard of always applying two-sided tests, although it would seem a one-sided test makes more sense here.

**Figure 5:**
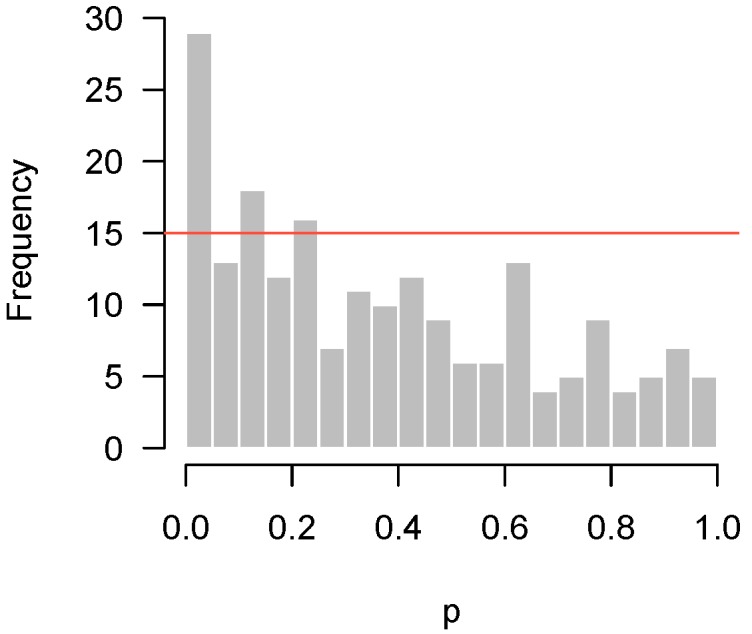
Rogier et al. [1]: Higher criticism threshold superimposed on Figure 3 indicating some genes may genuinely respond to the DSS.

Returning to our example from Rogier et al. [1] in Figure 3, b=0.05 and m=201, so $F_{0.95}\left(m,b\right)=15$. Figure 5 superimposes this threshold upon our earlier histogram. As the figure illustrates, the fact that 27 *p*-values fall below 0.05 provides significant evidence to reject the global null hypothesis, even though we cannot specifically reject any individual null hypothesis.

This is not a new idea in statistics, and dates back at least to John Tukey, who referred to this question as the “higher criticism” (HC) [4]. Tukey proposed the following test statistic, based on a normal approximation to the binomial:

$${\mathrm{HC}}_{0.05}=\sqrt{m}\left\{\frac{x}{m}-0.05\right\}-0.05,$$

where *x* is the number of *p*-values that fall below 0.05. One may then reject the global null at a 5% significance level if HC > 1.645. This leads to a very similar threshold as the above method for large numbers of tests (for example, with m=1000 tests, the binomial threshold is 62 and the Tukey threshold is 63). We prefer the more accurate binomial threshold for our purposes here, but note that Tukey’s closed-form approach has advantages for theoretical study and has received renewed attention in the past decade in the field of high-dimensional data analysis [5,6,7,8,9].

In particular, one limitation of the procedure we describe above, and of *p*-value histograms in general, is that they depend on an arbitrarily chosen bin width (in this paper, we use 0.05 throughout). To address this shortcoming, Donoho and Jin [5] considered an adaptive variant of higher criticism that involves varying the HC threshold to detect where the HC statistic is maximized. Similarly, it is widely recommended to vary the bin width when using histograms to visualize a distribution. With *p*-value histograms, this tends to be particularly relevant at the far left end of the histogram (i.e., in detecting patterns involving very low *p*-values). However, we have found that a bin width of 0.05 is in general a good place to start.

Thus, what should be made of situations such as that in Figure 5? Obviously, the main point of these sorts of experiments is to assess the veracity of individual hypotheses, and in that sense an experiment giving rise to Figure 5 must be viewed as unsuccessful. However, the higher criticism here implies that there is something to find—this experiment failed to find it, but another experiment, perhaps carried out with an improved experimental design or additional observations, might be successful. This is in contrast to the conclusion one would reach after looking at the histogram in Figure 1, which suggests that there is little hope in conducting another experiment investigating the same biological question, as there is simply nothing to find.

### 2.3. Quality Control

**Figure 6:**
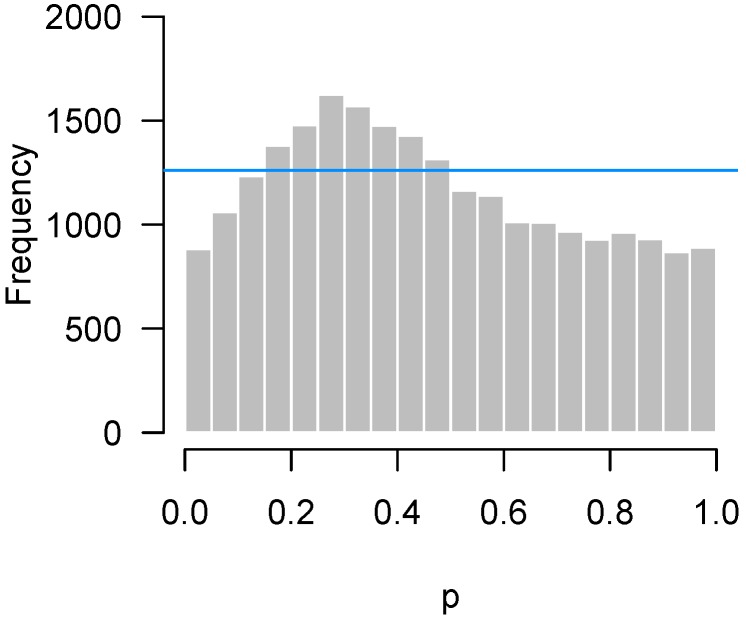
Fischl et al. [3]: Higher criticism threshold superimposed on Figure 4 indicates a possible problem in quality control.

The same basic idea can be used to test for departures from uniformity anywhere between 0 and 1, not necessarily only among low *p*-values. It is straightforward to extend the approach from Section 2.2 to this case using a Bonferroni correction. With a binwidth of 0.05, this amounts to checking 20 bins, and therefore using a corrected significance threshold of 0.05/20 = 0.0025, or equivalently, a frequency threshold of $F_{0.9975}\left(m,b\right)$. For the data from the study by Fischl et al. [3] in Figure 4, m= 23,332 and b= 0.05, so the frequency threshold is 1261. In Figure 6, this threshold is superimposed on the original histogram.

**Figure 7:**
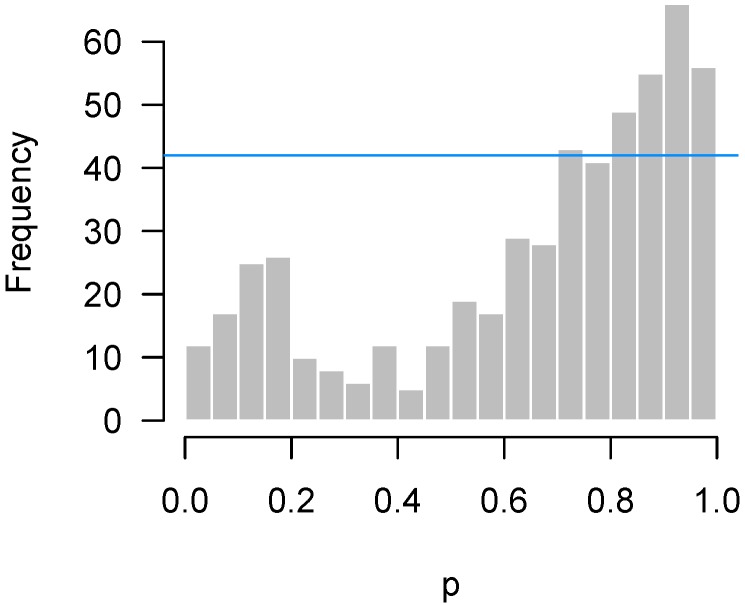
Bradley experiment: Indication of systematic deviation from theoretical null distributions.

As another example of an experiment whose *p*-value histogram displays a strange departure from uniformity, Figure 7 presents the *p*-values of an unpublished NanoString gene expression experiment conducted in 2012 by Dr. Luke Bradley at the University of Kentucky. These *p*-values were extracted from a two-way interaction effect in a three-way ANOVA model for each gene.

This procedure and the bound we have described are useful as a test of quality control, which we define broadly to mean any unexpected departure of the test from its distribution under the null hypothesis (notably, not including an abundance of low *p*-values, which would be expected). Here, it indicates that the excess of *p*-values around 0.3 in Figure 6 and the excess of *p*-values around 1 in Figure 7 are not due merely to random chance, but that some systematic deviation from the theoretical null distributions of the test statistics has occurred.

**Figure 8:**
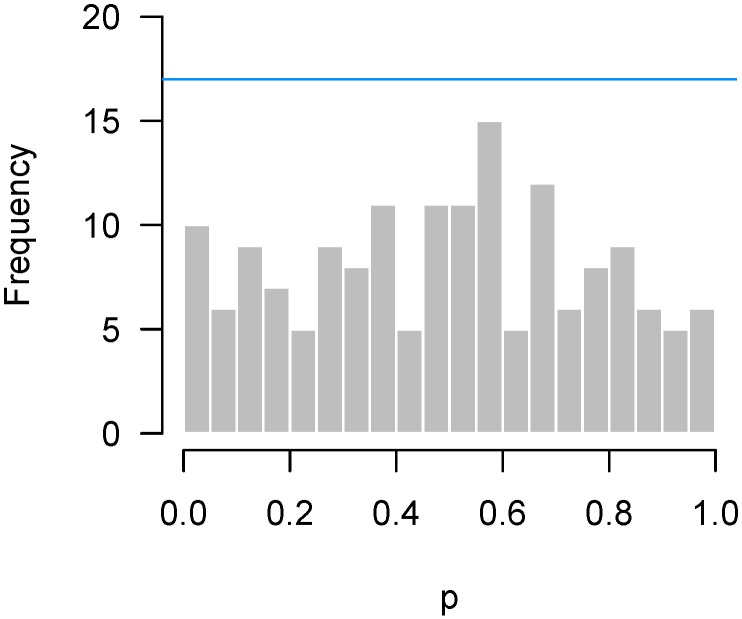
Matthews and Bridges [10]: Abundance of *p*-values near 0.6, although higher criticism bound suggests that this is likely due to chance.

In contrast, Figure 8 presents results from an experiment by Matthews and Bridges [10], in which steers were assigned randomly to graze either in a pasture that contained high levels of ergot alkaloids (n=10) or one that did not (n=9). The *p*-values come from a two-sample *t*-test of gene expression levels in the liver of the two groups of steers, as measured by NanoString. Although there is something of an abundance of *p*-values near 0.6, this excess is well within the bounds of random variation.

One drawback of the proposed quality control test is that it depends on an arbitrary bin size and is only looking for unimodal departures from uniformity. An alternative approach free of bin-width dependence would be to use a Kolmogorov–Smirnoff (KS) test of the *p*-values against a uniform reference distribution. In addition, the KS test is more powerful at detecting multimodal departures from uniformity. The main appeal of the quality control test we describe here is that it provides a helpful visual cue with which to interpret the *p*-value histogram and diagnose potential problems.

### 2.4. Causes of Anomalous p-Value Histograms

In this section, we explore some of the potential causes of the anomalous *p*-value histograms we have shown above. A related discussion is given by Brad Efron in Section 5 of Efron [11] and Chapter 6.4 of Efron [12]; we hope to add to Efron’s remarks by providing specific instances of these violations to illustrate the connection between the cause and the resulting shape of the *p*-value histogram. In Section 2.4.1 and Section 2.4.2, we simulate m=10,000 features belonging to two groups and use a two-sample *t*-test to test the null hypothesis that the means of the two groups are the same.

#### 2.4.1. Low Power

**Figure 9:**
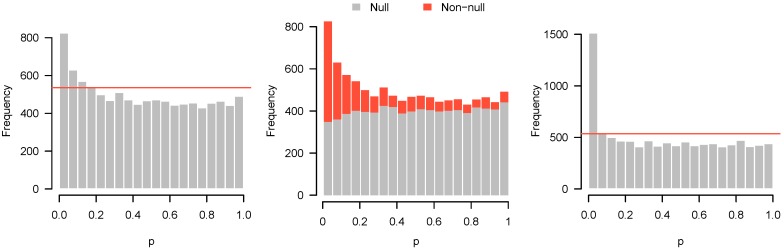
(**Left**) Simulated data with low power; (**Middle**) same data in(**Left**), showing contributions from null and non-null genes; and (**Right**) data simulated under same conditions as (**Left**), but with adequate power.

Here, we simulate n=4 observations in each of two groups from the standard normal distribution. For 80% of the features, there is no difference in the means. For the remaining 20%, the difference in means was drawn from a Uniform (−2, 2) distribution. The *p*-value histogram and accompanying higher criticism threshold are shown in Figure 9 (left).

With n=4, there is insufficient evidence to reject any of the individual null hypotheses, even at a liberal FDR cutoff of 30%. Nevertheless, the higher criticism threshold indicates that some of the features are non-null. Figure 9 (middle) shows a decomposition of the *p*-value histogram, revealing the contributions from the null and non-null features. As one might imagine from the shape of the histogram, the rise on the left side results from the fact that most of the non-null features have low *p*-values.

However, this is not true for all of the non-null features. With insufficient power, many of the non-null features turn out to have moderate, or even large *p*-values and can be found throughout all bins of the histogram. Obtaining these results is likely to be disappointing, since no significant features could be detected, but the *p*-value histogram and higher criticism indicate reasons for optimism. Although the initial experiment was unable to distinguish null and non-null features, there are indeed interesting features to be discovered, and a second, more adequately powered experiment may be successful at finding them.

To illustrate this, we simulated data under the same settings as above, but with a sample size of n=10 in each group. In marked contrast to the previous results, we can now safely reject 504 null hypotheses at the 5% FDR level. These results are displayed in Figure 9 (right), and show much clearer separation between null and non-null features.

#### 2.4.2. Incorrect Distributional Assumptions

**Figure 10:**
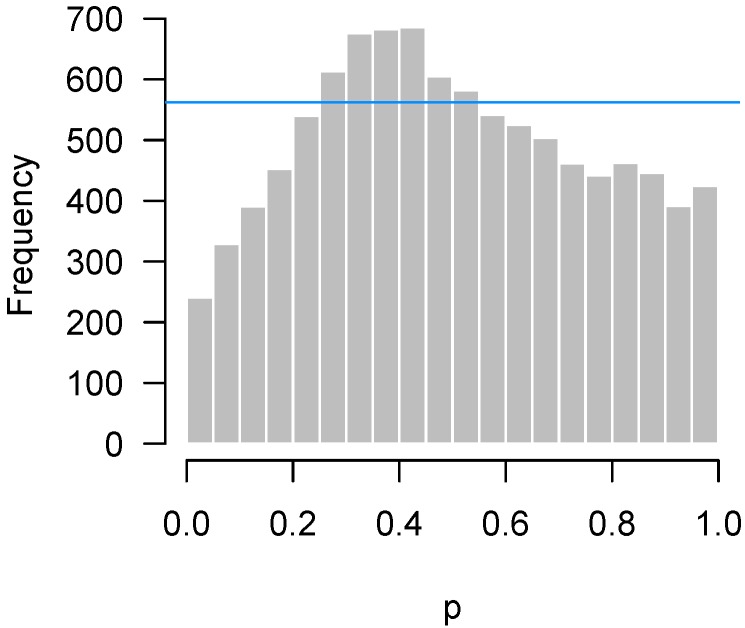
Simulated data: A *t*-test was applied, even though the data come from an exponential distribution.

In Figure 10, we simulate n=3 observations in each of the two groups from the exponential distribution with rate 1, and then apply a two-sample *t*-test for each feature. Thus, in this example, all 10,000 features satisfy the null hypothesis. The derivation of *p*-values from the *t*-test assumes normally distributed data; here, that assumption is highly inaccurate, the exponential distribution being both highly skewed and having considerably thicker tails than the normal distribution.

Problems with distributional assumptions can be alleviated by choosing more robust, nonparametric methods. For example, replacing the *t*-test in the above example with a Wilcoxon rank sum test produces an appropriate, uniform-looking histogram. In addition, distributional problems are alleviated as *n* increases due to the central limit theorem. Increasing *n* to 30 in each group for this setting also yields a flat, uniform-looking histogram essentially indistinguishable from Figure 1.

#### 2.4.3. Correlation among Features

Perhaps the most common cause of irregular-looking histograms, however, is correlation among features. With respect to *p*-value histograms, correlation among the features being tested does not necessarily alter the shape of the histogram: marginally, each *p*-value still follows a uniform distribution under the null. However, it does mean that there is a greater chance of seeing an irregular deviation from uniformity in the *p*-value histogram. For example, imagine a bundle of highly correlated features. Due to the correlation, these features will have similar *p*-values. Where the bundle lies is uniformly distributed, but, wherever it lands, a “bump” will appear in the histogram.

The higher criticism and quality control bounds in Section 2.2 and Section 2.3 are based on the assumption that the features being tested are mutually independent of each other. The primary practical consequence of correlation among features is that that the QC bound given in Section 2.3 is too low, leading one to conclude that an error has occurred when the irregular shape may simply be explained by correlation among the features.

Fortunately, given an adequate sample size, it is possible to assess the impact of correlation among features using permutation approaches. The idea underlying the permutation approach is simple. Let X denote the n×m matrix of feature values (here, gene expression data), with each row of X denoting an experimental unit consisting of *m* features. By permuting the rows of X, we accomplish two things. First, we eliminate any association between X and any other variables or group memberships that we are testing for. Second, by permuting entire rows of X intact, we preserve any correlation among the rows that is present in the data. Thus, by carrying out the original test on random permutations of X, we obtain *p*-values from the null distribution but without assuming independence among features.

**Figure 11:**
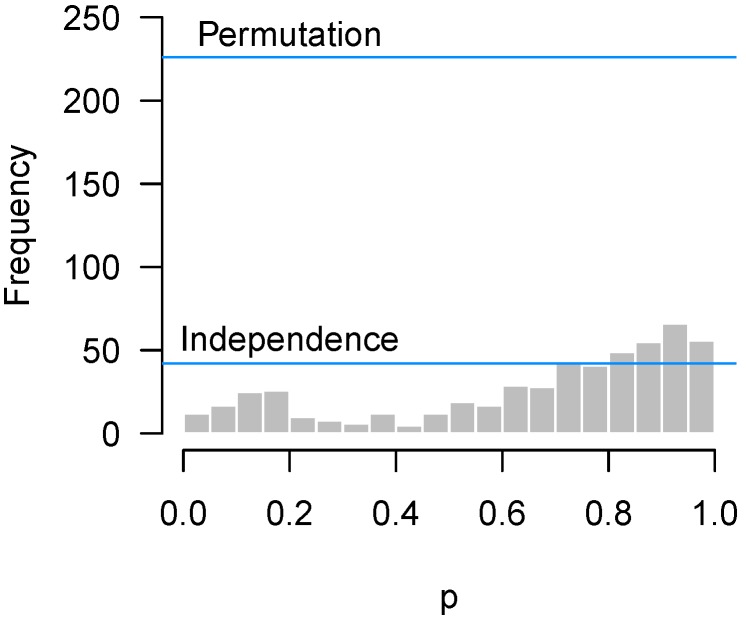
Bradley experiment: Permutation vs. independence approaches when the correlation between genes is high.

We repeated the test for the two-way interaction in the Bradley data seen in Figure 7 for 1000 random permutations of the expression data. For each permutation, we made a *p*-value histogram and recorded the count in the most highly populated bin. Figure 11 plots the histogram of the original *p*-values with two lines superimposed. One is the original quality control metric from Section 2.3 which assumes independence among the hypothesis tests, the other is the 95th percentile of the maximum counts from the permutation histograms.

The difference between the lines is striking. In this experiment, the correlation between genes is quite high (root-mean-square correlation among the 536 genes selected for the NanoString experiment was 0.75). As a result, the spike of *p*-values near 0.9 observed in the data could easily have arisen simply from the correlation among genes. In fact, given the correlation among features, the irregular-looking histogram of Figure 7 is not particularly unusual at all, a point clearly communicated by the large gap between the *p*-value histogram and the “Permutation” line in Figure 11.

Correlation among features also affects the higher criticism threshold of Section 2.2, although not as much as for quality control thresholds. The same permutation approach can be applied to obtain correlation-adjusted higher criticism thresholds, although in this case we would examine the 95th percentile of the counts for the first bin rather than the maximum count. For the Rogier et al. [1] data of Figure 5, the higher criticism bound assuming independence was 15, while the higher criticism bound obtained from the permutation approach was 19.4. This is far less dramatic than the difference in Figure 11 because, while correlation leads to bumps in the *p*-value histogram, those bumps are not systematically located in the lowest bin.

Unfortunately, there are limitations to the permutation approach. One is that it can be computer-intensive if *p* is large or if the tests themselves are time-consuming to perform. The other issue is that permutation approaches cannot be applied to very small samples. For example, we cannot use a permutation approach to investigate the Fischl et al. [3] data in Figure 6, which involves a one-sample *t*-test with only three pairs of subjects. Although the idea can be extended to paired data (by randomly assigning signs to the differences rather than permuting rows), in this case, there are only four distinct random assignments that can be made, and hence four different null histograms to serve as a reference for comparison, which is not sufficient for estimating a 95th percentile.

This is a fundamental limitation with applying permutation approaches to small samples, although the number of available permutations rapidly increases with sample size. For example, in a two-sample study with n=3 in each group, only 10 distinct permutations are available; however, with n=10 in each group, the number of permutations increases to 92,378.

For both reasons (small sample sizes and computational burden), it is desirable to develop an analytic method for estimating higher criticism and quality control thresholds that account for correlation among features. Such a development is beyond the scope of this manuscript, but we re-examine this issue in the discussion.

### 2.5. Remedies

When faced with an irregular *p*-value histogram, what action should a researcher take? In this section, we describe possible remedies.

One potential remedy is to increase the sample size by collecting more data. This is most clearly indicated in situations such as in Figure 3, where there is a clear indication that non-null features are present, but unable to be reliably distinguished from noise. The higher criticism threshold is potentially a very useful tool to guide this decision in terms of whether the additional cost of collecting more data is likely to bear fruit or not.

Alternatively, irregular *p*-value histograms may serve as an indication that the assumptions being made in the statistical analysis are not being met (see Section 2.4.2) and that one should consider an alternative approach—for example, a Wilcoxon rank sum test instead of a two-sample *t*-test. It is worth noting that higher sample sizes are beneficial here as well. Not only do larger sample sizes increase the robustness of many statistical tests, they also allow one to fit less restrictive statistical models.

Lastly, we note that irregular *p*-value histograms may also indicate that the experimental design should be revised. Although to some extent correlation among features is an unavoidable biological fact, it is also the case that careful experimental designs (randomization, blocking, balance, etc.) reduce this correlation and the potential for confounding factors to induce correlation in an experiment.

An element of design particularly relevant to expression and other sorts of “-omic” data is the issue of normalization. Proper normalization procedures substantially reduce correlations in this sort of data [13]. However, while normalization procedures are well-developed for long-standing technologies such as microarray data [14], this is often not the case for more recent technologies such as NanoString; normalization for RNA-Seq data has greatly advanced over the past decade, although developments continue to be made [15,16].

## 3. Discussion

### 3.1. Alternative Approaches

A common alternative to the *p*-value histogram is the QQ (quantile–quantile) plot, which plots the observed quantiles of the *p*-values against the expected theoretical quantiles of, in this case, a uniform distribution. The general idea of the plot is that if there is agreement between the observed and theoretical quantiles, the points will fall on the $45^{\circ }$ identity line.

The biggest difference between QQ plots and histograms is that QQ plots do not involve binning and can therefore reveal more information about individual tests than a histogram, especially at the extremes of the distribution. For example, suppose that we conducted 1000 tests, three of which had a very low *p*-value. The three significant results would be easily seen in a QQ plot, but could be obscured in the histogram.

For the most part, however, QQ plots and *p*-value histograms convey similar information regarding power and systematic discrepancies between assumptions and test results. The issues that we have described here could also have been detected by inspecting QQ plots; conversely, except for the example given above, anything one could learn from a QQ plot, one can also see in the *p*-value histogram. Which approach is better largely comes down to familiarity. An analyst familiar with interpreting QQ plots may see little benefit in *p*-value histograms, but many people, especially outside statistics, find QQ plots foreign and abstract, and are much more familiar with histograms. Much has been written about how to interpret QQ plots, but relatively little on *p*-value histograms.

Another alternative worth mentioning is to construct a histogram of test statistics rather than *p*-values. In this case, one would compare the resulting histogram to, e.g., a *t* distribution rather than the uniform. The primary advantage of plotting test statistics is that one can separately visualize each tail. For example, there may be a large number of overexpressed genes in an experiment but no underexpressed genes; such an asymmetry would not be apparent from a *p*-value histogram (assuming the *p*-values are from a two-tailed test). The primary disadvantage of plotting test statistics is that it is harder to see differences in the tails such as a subtle but consistent excess of low *p*-values.

**Figure 12:**
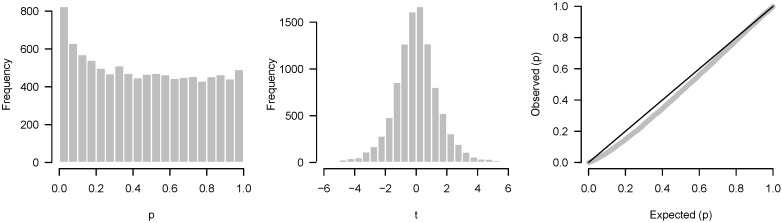
(**Left**) *p*-value histogram; (**Middle**) test statistics histogram; (**Right**) quantile-quantile plot.

Examples of these three plots, each applied to the data in Figure 9, are shown side-by-side for comparison in Figure 12.

### 3.2. Summary

In this article, we have taken a closer look at *p*-value histograms with respect to two questions of vital practical importance:

- Higher criticism: Is there a significant excess of low *p*-values? In other words, is there any evidence of a systematic biological response in the experiment?
- Quality control: Has something gone wrong in this experiment?

We present straightforward analytic diagnostics to address these questions, as well as a permutation-based approach capable of accounting for correlation among features. As Figure 11 demonstrates, correlation among features is an important issue as it has the potential to dramatically affect *p*-value histograms.

Our derivation of higher criticism bounds in Section 2.2 and quality control bounds in Section 2.3 assumes that the *p*-values are proper (see Section 2.1). Many common tests, however, especially those involving discrete outcomes, are valid but not proper. For these conservative tests, the higher criticism derivation still holds, although similar to the tests themselves, the threshold will be conservative. However, for the quality control bound, this issue causes a problem, as a bump in the histogram could be the result of the conservative nature of the test and not an actual problem with the experiment. The quality control bounds derived in Section 2.3 are not likely to be useful for such tests, although the permutation approach may still be used.

An additional factor that can distort *p*-value histograms, but which is not discussed in Section 2.4, is the effect of correlation among sampling units, possibly brought on by unmeasured confounding variables. The effect of correlation among samples (as opposed to correlation among features) is to broaden the null distribution. If this correlation is not accounted for, it will lead to an inflation of test statistics and a failure to preserve the proper size of the test, rejecting the null hypothesis too often. This is obviously an important issue, although *p*-value histograms are of little help in diagnosing this issue, since, when this issue is present, the histogram appears similar to “ideal” results, with a clear excess of small *p*-values.

Finally, as noted in Section 2.4.3, it is desirable to develop an analytic method capable of computing higher criticism and quality control thresholds without the need for a permutation approach. Such a method, however, would need to both estimate and account for all pairwise correlations among the features. This is potentially a very large number, especially for genome-wide expression studies. These statistical challenges are not necessarily insurmountable, but they do fall beyond the intended scope of this article.

Despite these limitations, it is our hope that the tools and examples presented in this article will be useful to researchers engaged in testing of high-throughput biological data, particularly since advice on “troubleshooting” such experiments is difficult to find in the scientific literature as problematic and underpowered studies often go unpublished.

## Appendix A

The histograms can easily be reproduced in R (www.r-project.org) with the following code, which assumes that a vector p of *p*-values has already been calculated:

b **<**- 0.05
**hist**(p, breaks=**seq**(0, 1, b), **col**=“gray”, border=“white”)
# *Higher criticism:*
**abline**(h=**qbinom**(0.95, **length**(p), b), **col**=“red”)
# *Quality control:*
**abline**(h=**qbinom**(1 − b*0.05, **length**(p), b), **col**=“blue”)
